# Supplementary material for: Identity-by-descent with uncertainty characterises connectivity of Plasmodium falciparum populations on the Colombian-Pacific coast
Source: PLoS Genet. 2020 Nov 16;16(11):e1009101. doi: 10.1371/journal.pgen.1009101 (PMC7704048; doi:10.1371/journal.pgen.1009101)
Supplement: S1 Table — (PDF) [file pgen.1009101.s001.pdf]

City (Province)	1993	1994	1997	1999	2000	2001	2002	2003	2004	2005	2006	2007	Total
Tumaco (Nariño)	0	0	0	2	2	10	11	59	0	23	0	25	132
Guapi (Cauca)	0	0	0	1	1	0	0	66	0	0	0	0	68
Buenaventura (Valle)	4	1	0	5	0	0	0	0	12	15	10	0	47
Quibdó (Chocó)	0	0	2	0	6	1	0	0	14	6	13	22	64
Tadó (Chocó)	0	0	0	0	0	12	2	0	0	0	0	0	14
Total	4	1	2	8	9	23	13	125	26	44	23	47	325
