# Supplementary material for: Identity-by-descent with uncertainty characterises connectivity of Plasmodium falciparum populations on the Colombian-Pacific coast
Source: PLoS Genet. 2020 Nov 16;16(11):e1009101. doi: 10.1371/journal.pgen.1009101 (PMC7704048; doi:10.1371/journal.pgen.1009101)
Supplement: S2 Table — Date and city refer to the date and city of collection of the earliest parasite sample per clonal component. (PDF) [file pgen.1009101.s002.pdf]

CC	Sample count	Longevity (days)	Date†	City†
CC1	13	2997	1999-03-15	Guapi
CC2	4	1935	1999-04-13	Buenaventura
CC3	2	49	2000-04-13	Quibdó
CC4	2	344	2000-06-29	Quibdó
CC5	9	1071	2000-11-23	Tumaco
CC6	8	1752	2001-01-29	Tumaco
CC7	3	399	2001-02-08	Tumaco
CC8	3	212	2001-02-17	Tumaco
CC9	4	32	2001-06-07	Tadó
CC10	2	28	2001-06-08	Tadó
CC11	2	101	2001-12-03	Tadó
CC12	15	1919	2002-04-03	Tumaco
CC13	2	442	2002-04-03	Tumaco
CC14	17	1896	2002-04-04	Tumaco
CC15	10	1921	2002-04-05	Tumaco
CC16	20	315	2003-01-07	Guapi
CC17	2	9	2003-03-03	Guapi
CC18	4	35	2003-03-07	Guapi
CC19	2	953	2003-03-17	Tumaco
CC20	28	1565	2003-03-22	Guapi
CC21	6	178	2003-04-11	Guapi
CC22	5	1	2003-05-15	Tumaco
CC23	2	149	2003-05-20	Tumaco
CC24	3	146	2003-05-29	Tumaco
CC25	5	28	2003-09-08	Tumaco
CC26	2	5	2003-10-01	Tumaco
CC27	2	6	2003-10-03	Guapi
CC28	2	43	2003-10-06	Guapi
CC29	2	545	2003-10-07	Guapi
CC30	2	9	2003-10-21	Tumaco
CC31	3	5	2003-11-05	Guapi
CC32	2	36	2004-05-31	Buenaventura
CC33	2	199	2004-07-23	Buenaventura
CC34	3	1016	2004-08-24	Quibdó
CC35	2	70	2004-10-27	Quibdó
CC36	2	30	2004-11-10	Quibdó
CC37	2	26	2004-12-10	Quibdó
CC38	6	850	2005-02-28	Quibdó
CC39	2	69	2005-07-27	Tumaco
CC40	5	694	2005-07-28	Tumaco
CC41	5	302	2006-08-03	Quibdó
CC42	2	13	2006-08-22	Buenaventura
CC43	7	301	2006-09-05	Quibdó
CC44	2	163	2006-10-10	Quibdó
CC45	4	96	2007-03-22	Quibdó
CC46	3	83	2007-03-28	Quibdó
