# Supplementary material for: Identity-by-descent with uncertainty characterises connectivity of Plasmodium falciparum populations on the Colombian-Pacific coast
Source: PLoS Genet. 2020 Nov 16;16(11):e1009101. doi: 10.1371/journal.pgen.1009101 (PMC7704048; doi:10.1371/journal.pgen.1009101)
Supplement: S3 Table — Average relatedness to three decimal places between clonal components (CCs) 1, 12, 14, 20 and 40 with the maximum 2.5% end-point of the 95% confidence intervals per CC in parentheses. The maximum 2.5% end-point indicates that relatedness between C20 and C40 is not statistically distinguishable from zero, for example. (PDF) [file pgen.1009101.s003.pdf]

	CC1	CC12	CC14	CC20
CC12	0.712 (0.624)			
CC14	0.733 (0.695)	0.648 (0.535)		
CC20	0.000 (0.000)	0.000 (0.000)	0.000 (0.000)	
CC40	0.000 (0.000)	0.000 (0.000)	0.000 (0.000)	0.079 (0.000)
