# Supplementary figures and images for: Identity-by-descent with uncertainty characterises connectivity of Plasmodium falciparum populations on the Colombian-Pacific coast
Source: PLoS Genet. 2020 Nov 16;16(11):e1009101. doi: 10.1371/journal.pgen.1009101 (PMC7704048; doi:10.1371/journal.pgen.1009101)

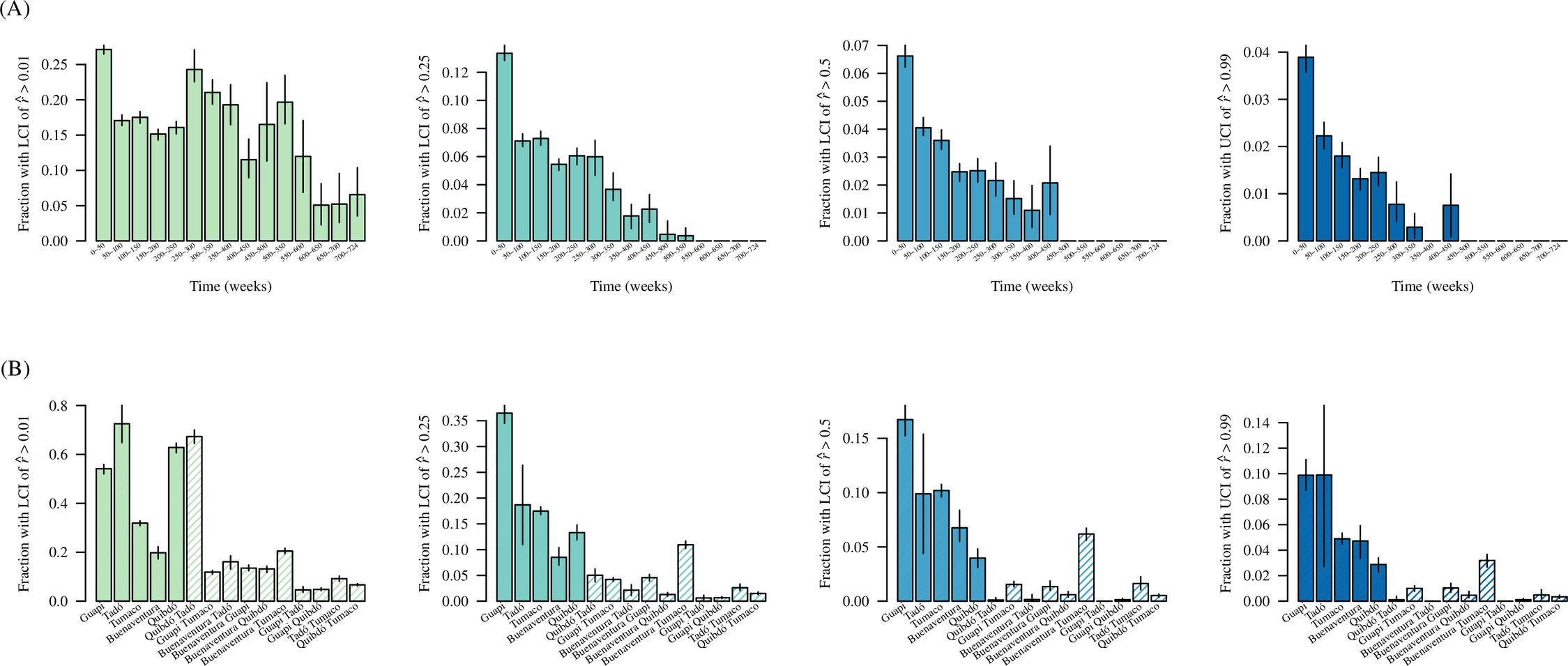

Supplement: S1 Fig — Highly-related samples pairs are defined as those with lower confidence interval end-point (LCI) of relatedness estimate, r^, greater than thresholds 0.25 and 0.50; or with upper confidence interval end-point (UCI) of r^>0.99 (i.e. clonal parasite sample pairs). Colours correspond to Fig 1. (A) Partitioned by time between collection dates. (B) Partitioned by collection city. (TIF) [file pgen.1009101.s004.tif]

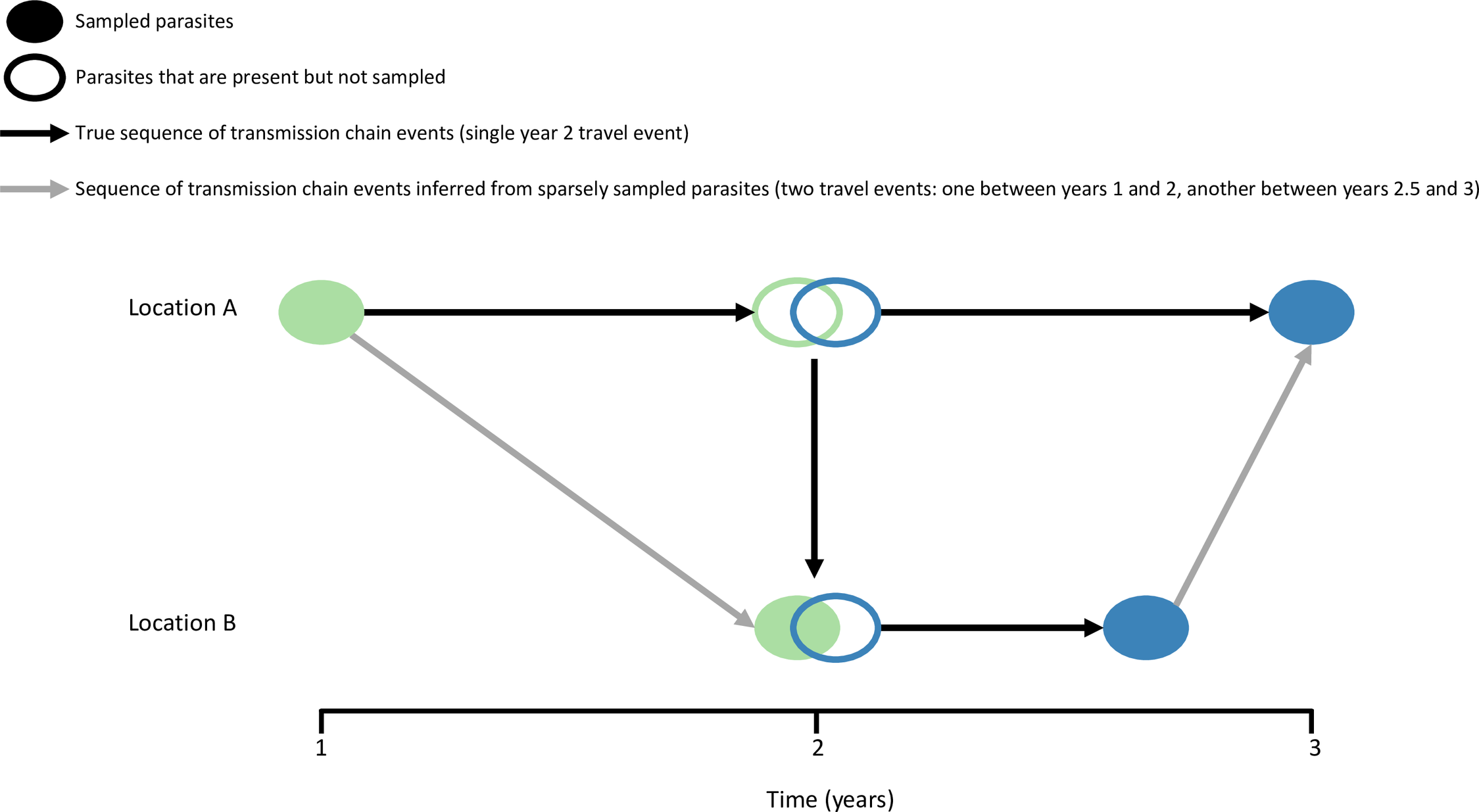

Supplement: S2 Fig — Schematic illustrating why sample collection chronology is not necessarily representative of the true sequence of transmission chain events when sampling is sparse and clonal propagation is frequent. The schematic shows two hypothetical locations A and B where malaria parasites have been sampled sparsely: solid ellipses represent sampled parasites, open ellipses represent parasites that were present but not sampled, different colours denote different parasite genotypes. (TIF) [file pgen.1009101.s005.tif]

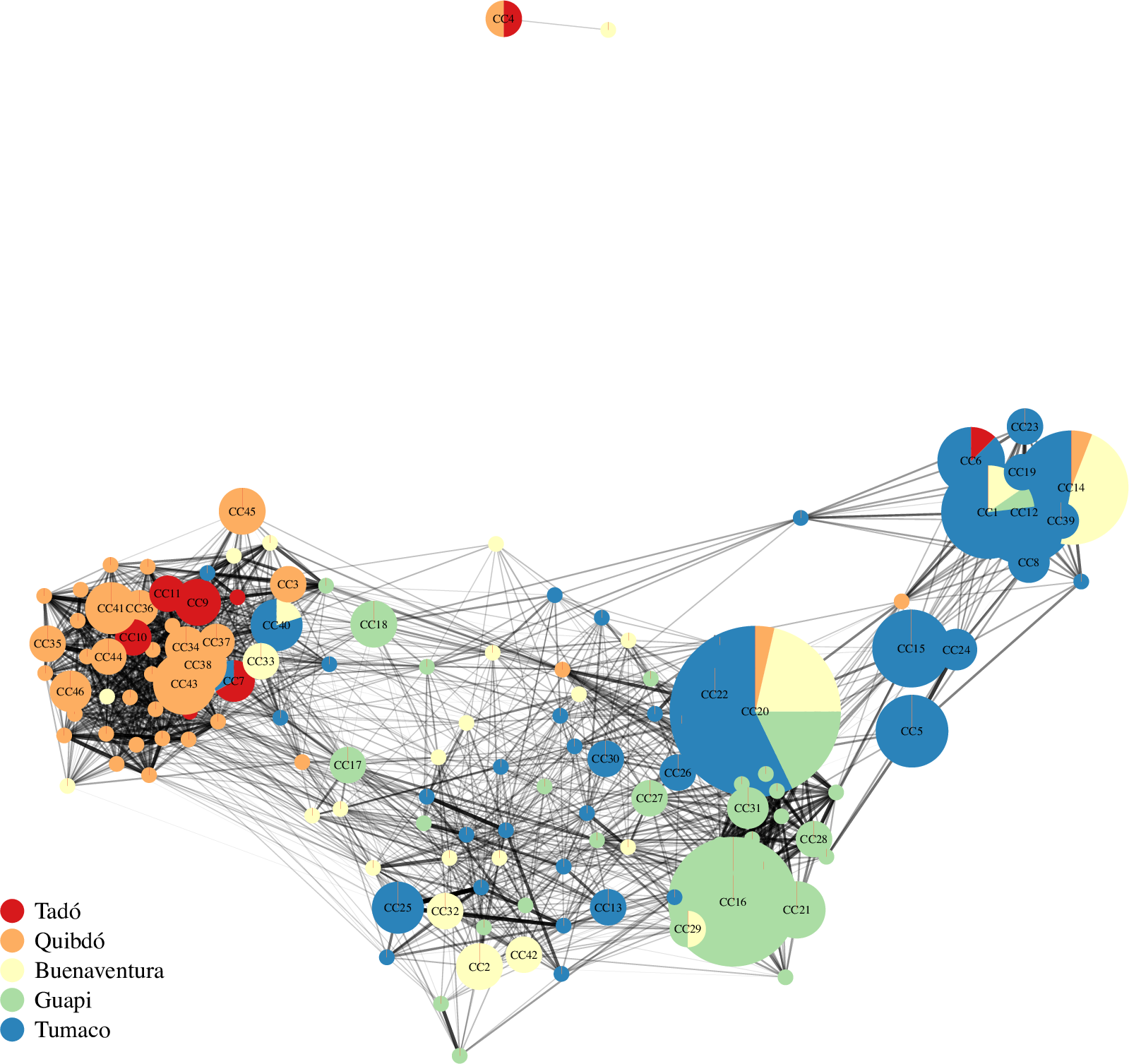

Supplement: S3 Fig — Vertices depict clonal components (CCs), which are groups of two or more statistically indistinguishable parasite samples, and singletons, which are individual parasite samples that do not belong to a CC. Vertices are plotted using the Fruchterman-Reingold layout algorithm [45], thereby clustering inter-related vertices. The size of each CC vertex is proportional to the number of parasite samples per CC, ranging from 2 to 28 statistically indistinguishable parasite samples. CCs are named in order of the collection date of the earliest parasite sample per CC. CCs with parasite samples from two or more sites are depicted as pie charts. Colour denotes the city of parasite sample collection. Edge transparency and weight is proportional to average relatedness, ranging from 0.003 to 0.912. Relatedness estimates that are indistinguishable from zero were set to zero. Edges whose average relatedness is zero are not plotted. Each CC besides CC4 is related to at least one other. CC4 is likely a contaminant; see main text. A singleton from Buenaventura, which is loosely related to CC4, may also be a contaminant. (TIF) [file pgen.1009101.s006.tif]

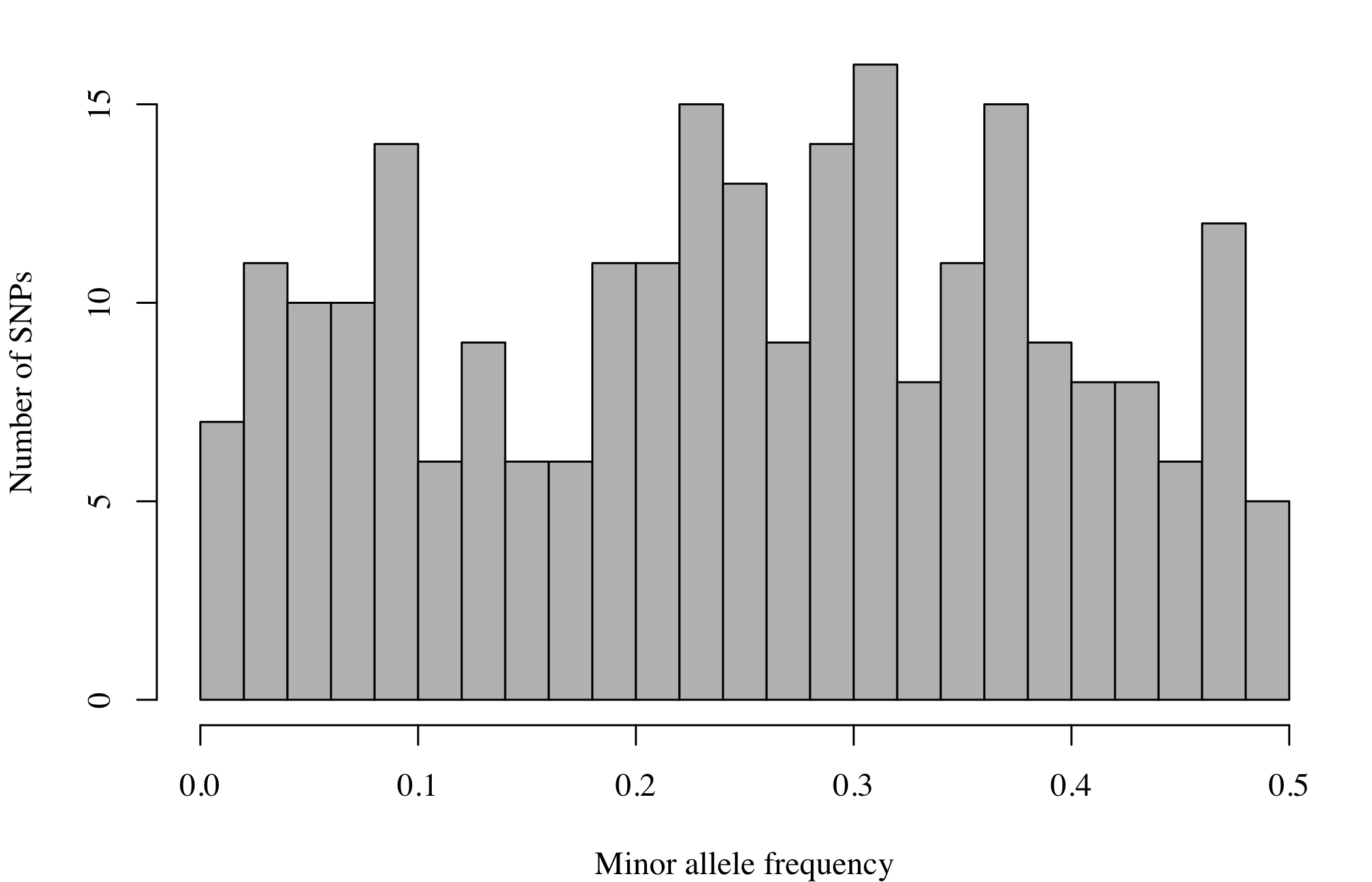

Supplement: S4 Fig — Histogram of minor allele frequencies estimated using all 325 monoclonal P. falciparum samples genotyped at 250 biallelic SNPs. (TIF) [file pgen.1009101.s007.tif]
