# Supplementary material for: Identity-by-descent with uncertainty characterises connectivity of Plasmodium falciparum populations on the Colombian-Pacific coast
Source: PLoS Genet. 2020 Nov 16;16(11):e1009101. doi: 10.1371/journal.pgen.1009101 (PMC7704048; doi:10.1371/journal.pgen.1009101)
Supplement: Attachment — Submitted filename: Response_to_reviewers.pdf [file pgen.1009101.s008.pdf]

Reviewer #1: The authors perform identity-by-descent (IBD) analysis on genotype data for 325 P. falciparum samples in order to understand Malaria transmission in five cities along the Colombian-Pacific coast. Results from this analysis compliment and expand on a previous study of this cohort which used alternative techniques to examine transmission along the coast. The authors provide evidence of greater diversity in P. falciparum genomes in cities with heavy marine traffic, as well as greater gene flow between cities with marine ports than those without.

Results from this analysis suggest a need for greater precautions at wharfs and ports along the Colombian-Pacific coast to reduce Malaria transmission via marine traffic. This is a well written manuscript that uses statistically sound and logical analyses, where the results are supported by geographical and marine traffic data. I also commend the authors on their original and informative graphics. I have only minor comments.

We thank the reviewer for their supportive feedback and the time they have taken to review our manuscript.

Minor comments

• In figures 5 and A.2, the samples from Tado and Quibdo are highly related within and between these two cities, even though there is ~3 years difference the collection date between the samples from Tado and Quibdo (e.g. CC10 and CC34). I assume the geographical location/isolation of Tado and Quibdo may explain the genotypic similarity. Would this also explain why the samples have remained so genetically similar over time, or could there be another explanation for this?

Yes, we agree - the geographic location and isolation of Tado and Quibdo is consistent with the clustering of vertices in Figs 5 and A.2. These cities are ~49 Km apart, about 1h 25 min of travel. Travel cost is 27.000 COP (7 USD). Every hour or an hour and a half, buses or taxis are in service to transport people between the cities (high human connection). This was confirmed by a Faculty of the Universidad tecnologica del Choco (located in Quibdo).

Although time between the collection dates of the first samples detected in CCs in Tado and Quibdo are long. The longevity of clones in these two sites is not unlike the longevity of clones in other locations; see revised supplementary Table A.4 and plot below, available from the revised code:
https://github.com/artaylor85/ColombianBarcode/blob/master/Code/Plot_graph_components.R

Otherwise stated, long-term clonal propagation is a general characteristic of malaria transmission on the Colombian-Pacific coast, which moreover extends southward to Ecuador (Sáenz et al. Malaria journal 14.1 (2015): 497 and Vera-Arias et al. Malaria journal 18.1 (2019): 251), both cited in the revised discussion).

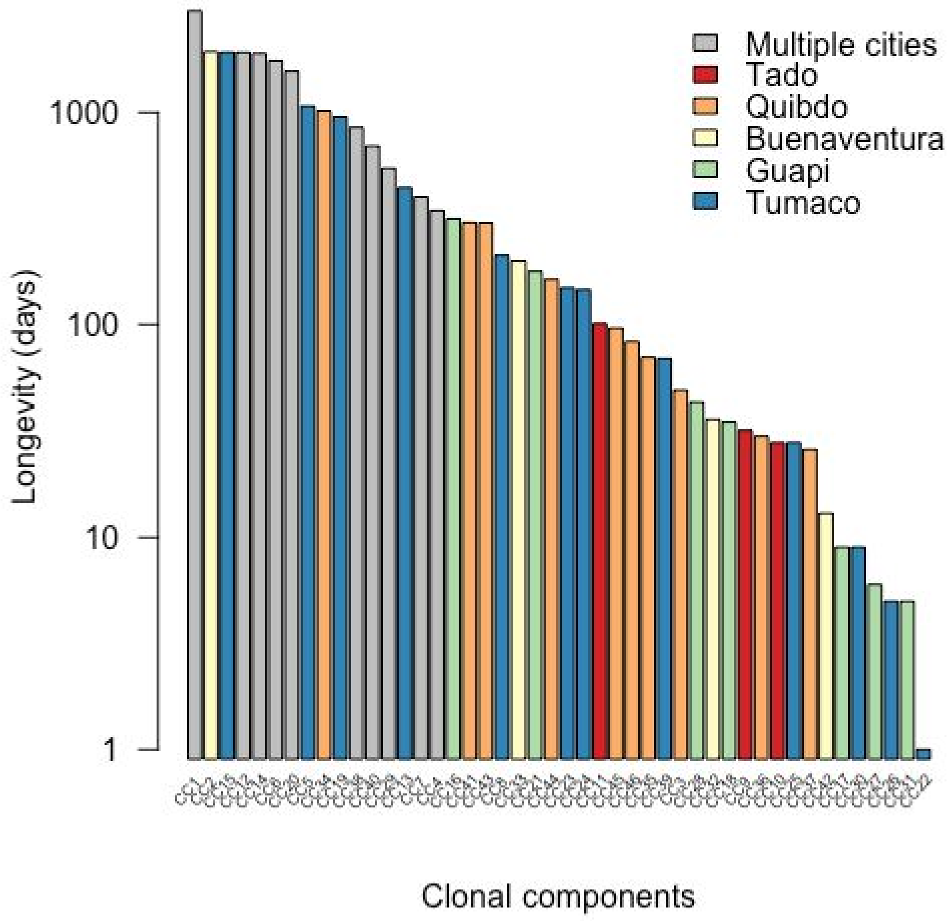

• Line 55 page 2: I cannot make sense of the following sentence, please re-write. “As such, analyses of relatedness can sometimes nearby and recent connectivity where analyses of FST cannot.”

Apologies for the confusion, a word was mistakenly omitted. We also note that this sentence could be misunderstood and thus have revised it. It now reads “As such, analyses of relatedness sometimes recover nearby and recent connectivity where analyses of FST do not.”

Reviewer #2: This is a well-written ms detailing the development and application of IBD relatedness approaches in malaria populations sampled along the Pacific Coast of Colombia. As the authors state relatedness approaches including those which utilise IBD approaches are widely used but rarely, to my knowledge, provide estimates of uncertainty. I would think that the methods described in the ms should be of relevance to a broad swathe of thr PLoS Genetics readership, particularly those interested in conservation biology/ genomic epidemiology in recombining organisms. Most of my comments are of a picayune/ muggen ziften nature with possible exception of comments starting "Line 149" and "Line 163."

We thank the reviewer for their comprehensive review and supportive feedback. Please excuse the latex formatting (e.g. \cite{reference} in the verbatim copied below. All changes have tracked using Latexdiff and are thus easily identifiable in pdf of the revised manuscript.

Line 41 distinct rather than disparate (a difference in essence) Done
Line 45 clarify which "them" Done
Line 55 something missing here Done: added "recover"
Line 60 "In any event" superfluous Removed

Line 63 Although transmission of distinct parasite clones could be the result of separate infection events over the course of the mosquito life span Yes, we agree this is a formal possibility. The revised text now reads. "Humans can be infected by multiple genetically distinct parasite clones that are either co-transmitted via inoculation from a single mosquito, in which case they are likely recombinants so inter-related (unless they derive from different blood meals)..."

Line 74 sentence "Departure…." Partial repeat of previous Removed

Line 77 solely in malaria epid or more generally We haven't seen any examples elsewhere neither, thus added "(and, to our knowledge, for the first time in ecological and epidemiological studies more generally)"

Line 79 curious phrasing "our response to it" Removed

Line 83 is it critical? Does drug policy change at a sub-national level? - Needs more support for such a broad statement Yes, we believe it is critical, as the following example, which has been added to the revised text, illustrates. For example, if resistance is driven by spread (versus de-novo mutation), targeted efforts to eliminate hotspots of transmission (e.g. in eastern Myanmar \cite{parker2017,landier2018}) may help to prolong the longevity of compromised antimalarial therapies. To ensure adequate isolation, thereby prevent re-population, units of targeted intervention need to account for parasite population connectivity, which relates to human migration \cite{blanton2018, wesolowski2018, gao2020}

Line 88 direction of clonality and incidence relationship- presume inverse? True - greater incidence is thought to cause greater clonality. However, the previous study reports a correlation only. We have thus reversed the order, but without implying causality. The revised manuscript now reads: "strong association between incidence and clonality".

Line 102 require a little more explanation of what data the threshold was based upon eg % SNPs shared in pairwise comparisons. Added: “For example, in \cite{taylor2017} a parasite pair was considered highly related if its relatedness estimate exceeded 0.5, whereas here a parasite pair is considered highly related if the lower bound of the 95% confidence interval around its relatedness estimate exceeds some stated value, which is 0.25 in the main text and 0.5 in sensitivity analyses.” (the threshold considering uncertainty is slightly more nuanced thus lengthy to explain, which is why we only alluded to it in the original text).

Line 106 “optimal transport using Wasserstein distance” will read as jargon to the casual reader (and to me) The revised text reads: “First, we complement our analysis of highly-related parasites with a threshold-free approach that uses a metric called the 1-Wasserstein distance, which can be interpreted as the cost required to transport a distribution of parasite samples from one city to another \cite{Feged-Rivadeneira2018, peyre2019}.”

Line 111 not clear what brittle means in this sentence The revised text reads: “Graph components circumvent reliance on unsupervised clustering methods that are sensitive to both the definition of genetic similarity and algorithmic specification \cite{pritchard2000, watson2020}.”

Line 115 something missing here “plan to”? Yes, added.

Line 118 State which relatedness estimator was used Done. The revised text reads: ”relatedness was estimated using the hidden Markov model (HMM) described in \cite{Taylor2019}. Relatedness is thus defined as the probability that, at any SNP, the two alleles drawn from the paired monoclonal \textit{P. falciparum} samples are IBD.”

Figure 1 more detail in figure legend required eg what’s tau? IN general I find the figure legends a little brief. Always feel that figures should be comprehensible without reference to the text Done
Table 1 similarly define r hat; epsilon etc Done

Line 124 capital H? Done

Line 125 define why is this a high relatedness threshold? The 0.25 threshold has a clear interpretation in an outcrossed population. However, in a population of malaria parasites that sometimes self, 0.25 is somewhat arbitrary (hence the inclusion of sensitivity and threshold-free analyses). The revised text reads: “highly-related parasite samples were classified using an arbitrary threshold of 0.25 (Table \ref{tab:classification}), which corresponds to the expected relatedness of parasites separated by two outcrossed generations, but is hard to interpret in the context of frequent clonal propagation.”

Figure 2 colour contrast in panel b is poor. Also font size of legend and axis labels too small. Increased contrast, font sizes of legend, axis title and label.

Is distance measured by simple straight line or by some other means eg via transport infrastructure Inter-city distance was measured by simple straight line. Specifically, the inter-city great-circle distance; see revised Figures 2 and 3. Great-circle distances were

calculated using the Haversine formula, which assumes the earth is spherical; see revised Methods section.

Line 128 replace far apart with space We mean to say close together versus far apart in time, not close together in time versus space. The revised text simply reads: “closer together in time”

Line 129 replace versus with than Done

Line 131 exceptionally high? Perhaps greater than expected given geographic distance Done. The revised text now reads “greater than expected given inter-city distance”

Line 135 meaning of “effort required” is unclear Changed “effort” to “cost” (here and in the methods); and added “where the cost of transporting a single parasite to another is equal to one minus relatedness” to the results section, and “Since per-city parasite sample sizes differ, transportation requires the expansion (or contraction) of parasite mass in addition to the transportation of individual units.” to the methods section.

Line 142 capitalise pan-american Done

Line 149 Can you test this supposition by analysing data within years or at least within the 2004-2206 collection window from which most samples derive to demonstrate elevated relatedness in temporally proximate collections? Yes, good idea. In the following, “this supposition” is taken to be “The low fraction of highly-related parasite sample pairs within Buenaventura (Figure 2b) is consistent with it having contributed samples over many years (Table A.2)”. Plots of the fraction of highly-related samples within Buenaventura per year suggest the sampling window for Buenaventura partly explains the lower fraction with the exception of 1993 (when there were only 4 samples, thus 6 comparisons) and 2004 (when there were 12 samples, thus 66 comparisons); see plot below, available in the revised code (https://github.com/artaylor85/ColombianBarcode/blob/master/Code/Generate_proportions_BuenaventuraOnly.R). We have thus added “in part” to the revised text. It is important to note that the overall proportion is not an average of the yearly proportions since overall inc. all comparisons within and across years (1081 in total) whereas yearly do not (232 in total - summed over yearly comparisons).

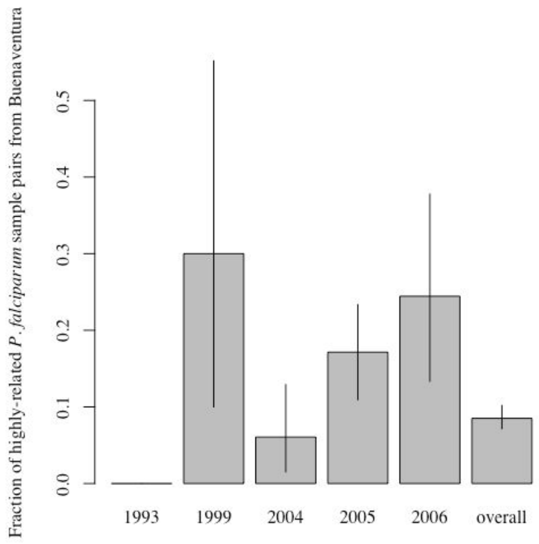

Line 160 I think a small expansion is required here rather than referring to methodology. This sentence cannot be readily parsed by the general reader Added “consider a graph whose vertices are parasite samples and whose edges are weighted by relatedness estimates, a clonal component is a sub-graph whose vertices are all connected to one another via edges whose weights are statistically indistinguishable from one (i.e. clonally related, Table \ref{tab:classification})”

Line 163 how is clonal relatedness defined We consider relatedness estimates that are statistically indistinguishable from one to be clonal – see Table 1, and have added “(i.e. clonally related, Table \ref{tab:classification})” to the definition of a clonal component above (see above revision).

Line 163 How can you discount a rare importation event from genetically distinct population? Obviously this putative contamination event was only detected as the clonal component was so different. I would imagine that contamination events at time of collection would inflate estimates of relatedness within cities and within time points. How would you refute this suggestion? In this specific case, we know CC4 is an artefactual contaminant because it maps to Dd2, a reference genome from Southeast Asia, which was included as a control when the data were originally generated; see revised text.

Considering importation more generally, we agree that detection depends on how genetically distinct imported parasites are. In general, however, importation is likely to deflate relatedness within cities and time points. Indeed, this is why we state “The low fraction of highly-related parasite sample pairs within Buenaventura (Figure \ref{subfig:space}) is consistent with […] it being the most important port on the Pacific coast (Figure \ref{subfig:marine traffic}), i.e. a hub through which human traffic and thus potential parasite mixing is high \cite{Echeverry2013}.”

Fig 3 same issue with font size Increased contrast, font sizes of legend, axis title and label.

Figure 4b perhaps overlay city labels? The zoom feature did not work on my paper print out. The sentence beginning “Zoom in” has been deleted – the map cannot be modified as doing so would breach the Google terms of service.

Line 173 would be good to see some data discussing MOI in these populations to support this assertion. We have revised the wording of the assertion. It now reads “As such they could derive from a single superinfected individual, or from different individuals with independent monoclonal infections.” Either way, to support this assertion data on the proportion of infections with MOI = 2, 3, etc. are required, as well as data on the relatedness between clones within multiclonal infections (e.g. single cell sequencing data of Nkhoma et al. 2020) to better understand the contribution of superinfection among multiclonal infections. Neither of these are available. We have added “Unfortunately, the data required to further evaluate these scenarios (data on the multiplicity of multiclonal infections, and on relatedness within multiclonal infections, e.g. [12]) are not available.” to the revised text. We can, however calculate the probability that the five clones shared between Buenaventura and Tumaco were distributed across infections in four or fewer individuals: let (1-0.5*(0.14 + 0.19)) be the probability of a monoclonal infection averaged across Buenaventura and Tumaco, such that (1 - 0.5*(0.14 + 0.19))^5 is the probability of all 5 infections being monoclonal, and 1-(1 - 0.5*(0.14 + 0.19))^5 = 0.6 is the probability of there not all five being monoclonal). This has been added to the revised text.

Figure 6 Not sure this is necessary I doubt anyone will dispute that without sampling to exhaustion not all haplotype will be detected in a single collection period Fig 6 has been moved to Fig A.2.

Line 196 verbatim repeat of sentence in introduction Adapted such that more concise and without duplicate reference to the many citations: “Our overall approach could be adapted for analyses of other recombining organisms with mixed mating systems.”

Line 226 “gold and narcotics” is this necessary? Could be seen to be perpetuating a stereotype. Would international trade suffice? Done

Line 229 Second sentence does not support the assertion that flow of infected migrants has increased, just reveals that they come predominantly form Venezuela. To support the first sentence and reveal that international migrants predominantly come from Venezuala, the revised manuscript now reads: “The flow of Venezuelan migrants infected with \textit{Plasmodium spp.} has increased in recent years: of 965, 1774 and 2288 non-domestic malaria cases reported in Colombia in 2017, 2018 and 2019, respectively, 882 (91.4\%), 1684 (94.9\%), and 2190 (95.7\%) were from Venezuela \cite{boletin2017, boletin2018, boletin2019}.

Line 236 Not trying to sanitise the ms but again “considerable violence” could this be rephrased- social instability—as written implies considerable violence is an innate property of the south pacific region rather than reflective of societal breakdown; malign foreign influence etc. Also why would high malaria case counts result in fleeting connectivity? Replaced by “social instability”. The reference to fleeting connectivity was unclear. The revised manuscript now reads: “The evidence we find of connectivity between \textit{P. falciparum} populations may be unique to the period of time over which the data

were collected (1993-2007). This was a period of historically high malaria case counts in Colombia \cite{rodriguez2011}, as well as social instability in the South Pacific region.”.

References are inconsistently formatted Done

Reviewer #3: This manuscript presents a nicely done reanalyses of a data set based on 13-27 year old samples from P. falciparum infection from the Pacific Coast of Colombia. Using the combination of IBD-based analytical approaches the authors demonstrated the importance of martime traffic in shaping the population structure.

While they may have not been applied in such a way to malaria epidemiology, the methods in themselves are not entirely novel. Similarly, why they very nicely and convincingly demonstrate the very particular nature malaria transmission in the study area, this does not lead to a fundamental change in our understanding local malaria epidemiology, rather it re-inforces patterns that have been previously recognised.

The authors claim broad relevance of their IBD-based approaches by pointing out that they could be applied other organisms with mixed mating systems. It would have been much more interesting if they had actually demonstrated that broad relevance by applying their methods either further, more recent malaria dataset (with different underlying transmission patterns) or to other organisms. Without such a demonstration, the manuscript remains just a nicely done case study of the power IBD-based analytical methods.

We thank the reviewer for considering our manuscript. However, we dispute the idea that this is just another case study of the power of IBD-based analytical methods; see revised abstract and discussion. While we do use IBD, we also make three important advances in support of our claim of broad relevance. For the first time in malaria epidemiology (and to our knowledge in ecological and epidemiological studies more generally):

1. We account for uncertainty around estimated relatedness. This is especially important considering a number of projects that plan to use data from panels of limited but diverse genetic markers (e.g. microhaplotypes) to do malaria genetic epidemiology. (Indeed, we plan to prospectively collect and jointly analyse data on both *P. falciparum* and on *Anopheles* spp. from South America, thereby applying our methods to more recent malaria data). This initial study provides a basis for those future analyses.
2. We use 1-Wasserstein distances to compare the cost of transporting parasite populations from one location to another, thereby comparing different parasite populations in an entirely threshold-free manner. This is especially important for analyses of recombining organisms with mixed mating systems because thresholds in these populations do not have clear interpretation (e.g. 0.5 may correspond to the expected relatedness of siblings in an outcrossed population, but its interpretation is less clear in a population where clonal propagation is common). We expect this initial study will serve as a starting point for further methodological studies of the application of concepts from optimal transport to genetic epidemiology.
3. We identify groups of parasite clones using graph components and confidence intervals, thereby circumventing reliance on an arbitrary clonal threshold, where arbitrary thresholds undermine the cross comparison of studies that used different data sets. Standardisation is a major goal in genetic malaria epidemiology, as it is needed to enable the translation of research into actionable insight for policy makers (Dalmat et al. Malaria journal. 2019;18(1):163.)
